# Ageing and cancer: a research gap to fill

**DOI:** 10.1002/1878-0261.13222

**Published:** 2022-05-21

**Authors:** Eric Solary, Nancy Abou‐Zeid, Fabien Calvo

**Affiliations:** ^1^ Fondation « Association pour la Recherche sur le Cancer » Villejuif France; ^2^ Faculté de Médecine Université Paris Saclay Le Kremlin‐Bicêtre France; ^3^ Gustave Roussy Cancer Center INSERM U1287 Villejuif France; ^4^ 555089 Université de Paris France

**Keywords:** ageing, biological age, cancer, interception, policies, research

## Abstract

The complex mechanisms of ageing biology are increasingly understood. Interventions to reduce or delay ageing‐associated diseases are emerging. Cancer is one of the diseases promoted by tissue ageing. A clockwise mutational signature is identified in many tumours. Ageing might be a modifiable cancer risk factor. To reduce the incidence of ageing‐related cancer and to detect the disease at earlier stages, we need to understand better the links between ageing and tumours. When a cancer is established, geriatric assessment and measures of biological age might help to generate evidence‐based therapeutic recommendations. In this approach, patients and caregivers would include the respective weight to give to the quality of life and survival in the therapeutic choices. The increasing burden of cancer in older patients requires new generations of researchers and geriatric oncologists to be trained, to properly address disease complexity in a multidisciplinary manner, and to reduce health inequities in this population of patients. In this review, we propose a series of research challenges to tackle in the next few years to better prevent, detect and treat cancer in older patients while preserving their quality of life.

## Graphical abstract

The complex mechanisms that cause ageing can generate multiple late‐onset diseases, including cancer. Recently, several mechanistic links between ageing and cancer have been reported. In order to complete geriatric assessment and guide therapeutic choices in elderly patients with cancer, biological measurement of the effects of ageing has been proposed. This review explores multiple remaining issues to be addressed in an effort to improve cancer care in elderly patients.

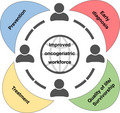

**Abbreviations**
BRCA1: breast cancer 1
CHEK2: checkpoint kinase 2
EC: European Commission
FGFR3: fibroblast growth factor receptor 3
NOTCH1: Notch receptor 1
PARP: poly(ADP‐ribose) polymérase
PPM1D: protein phosphatase, Mg2+/Mn2+ dependent 1D
SASP: senescence‐associated secretory phenotype
SMO: smoothened
TERT: telomerase reverse transcriptase
TP53: tumour protein P53

## 1 Introduction

During the last century, advances in medicine and sanitation have increased the average life span. A profound population transformation is ongoing. For the first time in human history, there are more people in the world over age 65 than under age 5. This trend, which has been apparent in more developed countries for several decades, is increasingly observed in less developed countries. By 2050, the number of people ages 65+ is projected to total just under 1.5 billion (16% of the world population). In 1950, it was only 5 per cent (https://www.prb.org/agingpopulationclocks/). There is simultaneously a deceleration of our perception of ageing effects, as people in their 80s nowadays resemble those in their 60s in the 1950s. A strong challenge for our health care system is to improve further healthy lifespan by reducing the pathological consequences of ageing.

Many of the mechanisms that cause ageing have been deciphered during the last four decades. These mechanisms appear to be rather complex, yet interventions directed at ageing are emerging, offering the opportunity to concurrently improve several late‐onset diseases [1, 2]. Together with cardiovascular, cognitive, degenerative and metabolic pathologies, cancer is one of the diseases that increases with tissue ageing in humans [3, 4]. The risk of suffering any cancer before the age of 40 is ~ 2%. By age 80, this risk increases to 50% and the incidence of most common cancers rises as a function of age [5]. The mechanistic links between cancer and ageing are beginning to emerge [3, 4, 5, 6]. By controlling the effects of ageing on cells and tissues, we might be able to both reduce the incidence of cancer and facilitate the treatment of ageing‐independent tumours by reducing the impact of comorbidities [1, 2].

**Fig. 1:**
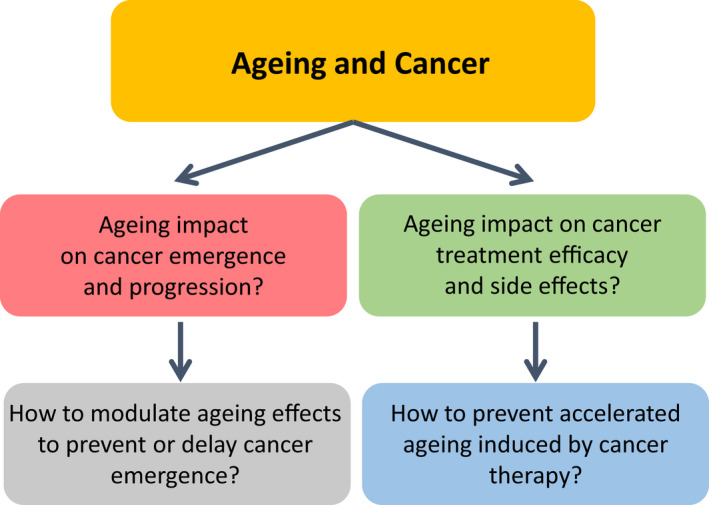
Main interactions between ageing and cancer.

The European Commission (EC) has identified cancer as one out of five mission‐oriented research and innovation topics in which researchers are challenged to deliver ambitious innovations that improve the quality of life of European citizens. The EC has also launched the Europe’s Beating Cancer Plan as a major political commitment to turn the tide against cancer with 10 flagship initiatives (https://ec.europa.eu/). The European population is ageing rapidly, and ageing is an essential risk factor for many cancer types in humans. Most human cancers arise in individuals over the age of 60, and the mean age of death from cancer is above 70. Therefore, interaction between ageing and cancer is a key area of intervention for the EC with two aspects, (a) to better understand how tissue ageing may promote cancer emergence and progression, and (b) to improve cancer treatment in older patients while preventing acceleration of ageing by cancer therapy (Fig. 1).

**Fig. 2:**
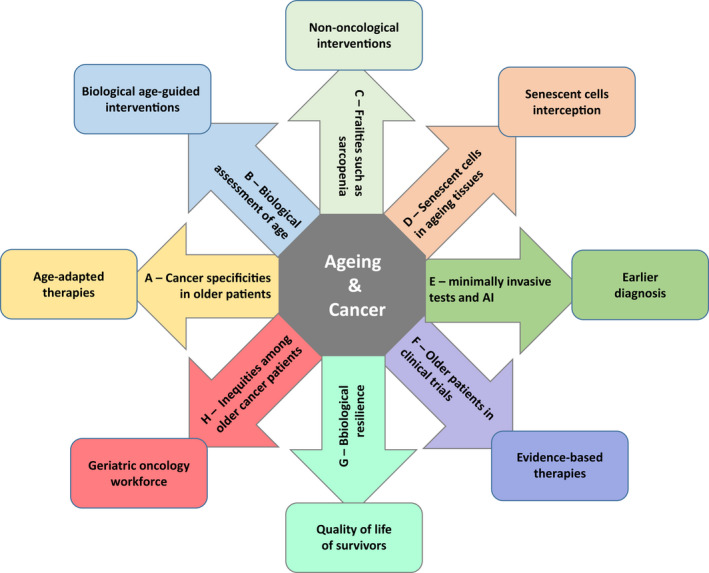
Eight areas of research on cancer interaction with ageing. The figure refers to the eight areas of research identified in this review. Arrows summarize the research field, and rectangles indicate potential interventions.

The translation of ageing research into cancer prevention and improved cancer care raises several difficulties [1, 2]. For example, ageing generates organ‐specific temporal signatures and thus may require tissue‐specific interventions. Nevertheless, the potential rewards of this translational approach should outweigh the difficulties. In this review, we propose a series of challenges that researchers, oncologists and geriatricians need to tackle together in order to better understand the interplay between ageing and cancer and to improve cancer prevention, cancer care and cancer survivorship in the growing population of ageing people (Fig. 2).

## 2 From ageing biology to cancer research

Cancer has long been described as the consequence of the multistep acquisition of somatically acquired DNA alterations, usually in stem cells, that lead to a growth advantage. Human cancers harbour hundreds to several thousand of mutations, a minority of which occur in recurrently mutated cancer genes and which drive mutated cell expansion into a clone. Recently, however, deep mutational screens of phenotypically normal human adult tissues have identified somatically mutated clones defined by well‐known drivers of carcinomas such as *NOTCH1*, *TP53*, *FGFR3* and *PPM1D* (List of abbreviated terms, Box 1) in otherwise healthy epithelial tissues, initially in the skin and the oesophagus [5, 7, 8, 9, 10]. Importantly, the number and size of these clones progressively increase with age (reviewed in ref. [11]) and virtually all renewing tissues become a patchwork of mutated clones over the time [12, 13]. Thus, mutations in oncogenic drivers are under positive selection in normal tissues. The accumulation of these somatically mutated clones might form part of the ageing process, which is commonly believed to occur due to the time‐dependent accumulation of cellular and tissue damage over time, which eventually lead to functional decline. The steady‐state accrual of some mutational processes during life is described as a molecular clock [14]. Exposure to environmental toxins increases the mutational burden in clones, as observed in the bronchial epithelium of tobacco smokers [15] and in the hepatocytes of cirrhotic patients [16].

Box 1 **Abbreviated terms**

***BRCA1*** (*Breast cancer 1*) gene encodes an E3 ubiquitin‐protein ligase involved in DNA repair. Constitutive mutations in BRCA1 and related genes predispose to breast and ovary cancer.

***CHEK2*** (*Checkpoint kinase 2*) gene encodes a serine/threonine‐protein kinase involved in cell cycle arrest, DNA repair and cell death in response to the presence of DNA damage.

***FGFR3*** (*Fibroblast growth factor receptor 3*) gene encodes a receptor involved in a wide array of pathways known to play a significant role in cancer.

***NOTCH1*** encodes a member of the NOTCH family of transmembrane proteins involved in cell fate specification, differentiation, proliferation and survival.

**PARP** (poly(ADP‐ribosyl)transferase) is a chromatin‐associated enzyme that modifies various nuclear proteins involved in DNA repair, cell differentiation and cell proliferation.

**PPM1D** (Protein phosphatase, Mg2+/Mn2+ dependent 1D) is a protein serine/threonine phosphatase involved in cell cycle regulation through the negative regulation of p53 expression.

**SASP** Senescence‐associated secretory phenotype defines the ability of senescent cells to secrete a variety of extracellular proteins and lipids.

***SMO*** (*Smoothened*, *frizzled class receptor*) encodes a G protein‐coupled receptor that interacts with the patched protein, a receptor for hedgehog proteins, and transduces intracellular signals.

**TERT** (*Telomerase reverse transcriptase*) is a ribonucleoprotein polymerase that, in progenitors and cancer cells, maintains telomere ends for the replication of chromosome termini in most eukaryotes.

***TP53*** gene encodes a tumour suppressor protein whose deregulation is detected in many tumour types.

**Fig. 3:**
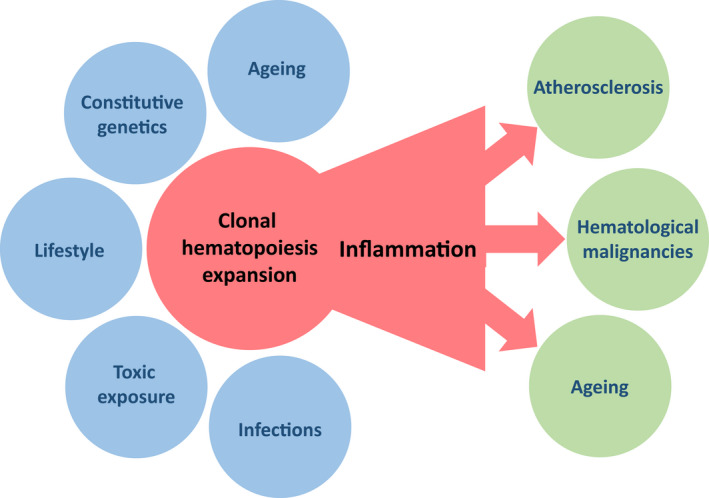
Evolution of clonal hematopoiesis of indeterminate potential (CHIP). The incidence of clonal hematopoiesis, as defined by the detection of one or several somatic mutations in at least 2% of circulating blood cells, increases with age. These clones can expand upon influence of multiple factors, including ageing itself, constitutive genetic background, lifestyle, toxic exposure and infectious diseases. Mature cells of the clone demonstrate a pro‐inflammatory phenotype. Their expansion can lead to an overt myeloid malignancy but also promote diseases such as atherosclerosis or accelerate ageing.

Fortunately, the mutant lineages identified in healthy tissues only rarely undergo transformation. This observation suggests that cancer may require a distinct evolutionary track from the one that expands these clones [17]. This is further indicated by the observation that some genetic variants found in oesophagus epithelium, such as *NOTCH1* mutations, confer improved fitness to normal cells, relative to cancer cells, indicating a context‐dependent effect of these variants [8]. Little is known about how the cell competition (Glossary, Box 2) in normal epithelia influences the early steps of cancer formation. In short, a tissue that accumulates damaged cells is at the same time an ageing tissue and a tissue at risk of developing cancer [4]. The accumulation of mutated clones with age, combined with other factors such as stromal cell senescence, may promote cancer development through diverse trajectories. A clone can toggle to a malignant tumour or create an inflammatory climate that, together with lifestyle‐related toxic insults, promotes the independent emergence of a cancer. In the hematopoietic tissue, clonal hematopoiesis of indeterminate potential (CHIP) generates pro‐inflammatory myeloid cells that can progress into overt myeloid malignancy or promote other diseases such as atherosclerosis or further enhance ageing hallmarks (Fig. 3). Interestingly, a recent and intriguing study suggests that mutant clones might also have an unexpected anti‐tumorigenic role through cell competition and preserve tissue integrity [18].

Box 2 **Glossary**

**Antagonistic pleiotropy.** As it applies to ageing, antagonistic pleiotropy indicates that animals might possess genes that enhance their individual fitness early in life but contribute to the ageing phenotype in later life.

**Biological age.** Also referred to as physiological age, biological age relates to decline in organ and tissue functions, independently of chronological age.

**Cell competition.** The process by which viable cells are eliminated from tissues, mostly epithelial tissues, by comparison with neighbouring cells.

**Chronological age.** Chronological ageing refers only to the passage of time, in other words to the actual amount of time a person has been alive.

**Clonal mosaicism.** Clonal mosaicism is the presence of one or more distinct populations of cells within an individual with an acquired genomic event that is not present in the inherited genome.

**Exposome.** This word refers to the measure of all the exposures of an individual in a lifetime and to how those exposures relate to health.

**Geriatric assessment.** A multidisciplinary process that identifies all the medical, functional and psychosocial limitations of a frail older person.

**Heterochronic parabiosis.** An experimental setting whereby an aged mouse and a young animal are joined surgically to reveal systemic regulators of ageing or age‐related diseases.

**Immunosenescence.** Ageing‐related changes in the immune system that contribute to the increased sensibility of ageing people to infections, auto‐immune diseases and cancer.

**Inflammaging.** A chronic, sterile, low‐grade systemic inflammation that develops with ageing and contributes to the pathogenesis of age‐related diseases.

**Machine learning.** A branch of artificial intelligence and computer science that uses data and algorithms to imitate the way that humans learn, gradually improving its accuracy.

**Neural networks.** A subset of machine learning based on the use of algorithms that recognize underlying relationships in a set of data through a process that mimics the way that biological neurons signal to one another in the human brain.

**Pre‐habilitation care.** A series of multidisciplinary health care interventions that aim to dampen side effects of medical or surgical therapies.

**Senolytic drugs.** Drugs that kill senescent cells selectively.

**Senomorphic drugs.** Drugs that delay the progression of young cells to senescent cells in the tissues or improve the functions of senescent cells.

**Survivorship.** In cancer, survivorship focuses on the health and well‐being of a person with cancer from the time of diagnosis until the end of life.

The presence of mutated clones in ageing but otherwise healthy tissues has blurred the frontier between noncancer and cancer clones. Why is it that the clones that accumulate with age do not generate multiple cancers remains poorly understood. Some might be cleared by terminal cell differentiation or by an active response in the surrounding tissues, involving immune cells [19] and nonimmune epithelial cells [20]. Whatever these clearance mechanisms are, their ability to efficiently eliminate mutated clones may decrease with age [8].

Telomere shortening and senescence might contribute to preventing tumour formation [21]. Telomere shortening typically limits the maximal number of divisions human cells can undergo. After a limited number of doublings, telomeres become very short and uncapped, which initiate DNA damage signalling and senescence [22]. Cellular senescence is a nonproliferative but viable state that is distinct from G0 quiescence and terminal differentiation [23]. Senescence was originally observed in normal diploid cells that cease to proliferate after a finite number of cell divisions (so‐called replicative ageing) [24]. Senescence is also a response to multiple stressful insults, including oncogenic stresses [25]. Cellular senescence is suspected to have both beneficial and detrimental effects and provides typical example of antagonistic pleiotropy (Glossary, Box 2). In young healthy tissues, mutated clones that enter replicative or oncogenic stress‐induced senescence are cleared away by surrounding cells. In ageing tissue, their clearance becomes less efficient, and senescent cells thus accumulate and disrupt tissue homeostasis through a variety of mechanisms. One such mechanism involves the development of a distinct secretory profile known as the senescence‐associated secretory phenotype (SASP) [21]. Senescence‐associated secretory phenotype creates a pro‐inflammatory milieu [an effect that is sometimes referred to as ‘tissue inflammaging’ (Glossary, Box 2)]. The decreased clearance of senescent cells by an ageing, disrupted tissue microenvironment may account for the development of a wide spectrum of age‐related diseases [22], including tumours [26].

When cells in which oncogenic changes accumulate escape senescence, they enter a state known as crisis. Cells that bypass this crisis usually engage telomerase reverse transcriptase (TERT) enzyme activity, which is absent in most healthy tissues and which is closely regulated in normal stem cells [27]. In most human carcinomas, telomerase is up‐regulated by diverse mechanisms. In other tumours, and especially in soft‐tissue sarcomas, an alternative mechanism can be activated, called the alternative lengthening of telomeres (ALT) [28]. Whatever the mechanism, cancer cells can be distinguished from nonmalignant clones by the reactivation of telomerase activity to bypass senescence. As an illustration, recent evidence from mice implicates TERT in setting the stage for the formation of pancreatic cancer [29]. Telomerase engagement may not be sufficient to generate a tumour, but telomere length maintenance might be required for the continuous growth of an established tumour.

Opportunities for early interventions are illustrated by analyses of hematopoietic tissue in which the presence of driver mutations generates clonal hematopoiesis in the blood of otherwise healthy individuals, the already mentioned CHIP. Some of these patients will subsequently develop one or several malignancies [30, 31, 32, 33, 34]. For example, *JAK2V617F*, a common driver mutation in myeloproliferative neoplasms (MPN), appears in a hematopoietic stem cell (HSC) very early in life, including *in utero*, and is followed by sequential driver events separated by decades during life, often outcompeting ancestral clones [35, 36]. It remains unclear how processes such as stem cell competition for niche occupancy influence the switch from a premalignant state to a malignant one. Of note, CHIP is strongly associated with the acceleration of multiple ageing‐associated clocks (Fig. 3) and may identify a human population that is at high risk of multiple ageing‐associated diseases and thus may be a target for clinical interventions [37, 38].

**Fig. 4:**
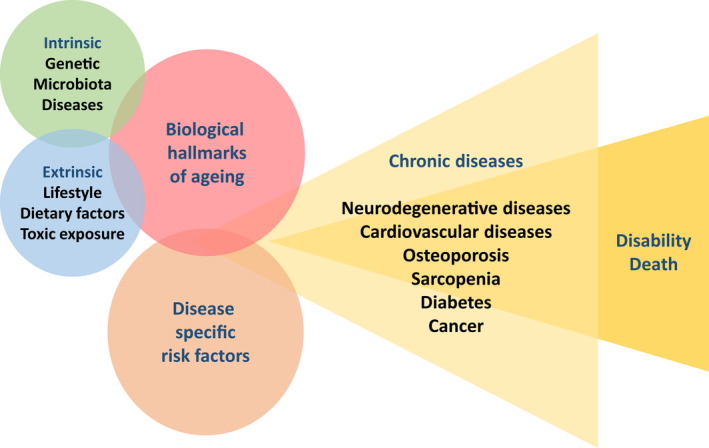
From ageing to disability and mortality. Intrinsic and extrinsic factors contribute to generate biological hallmarks of ageing in tissue cells. In combination with disease‐specific factors, tissue ageing promotes the generation of multiple chronic diseases including cancer, suggesting that a better control of ageing effects may prevent multiple diseases otherwise leading to disability and death.

Current evidence highlights the need for us to better understand the relationship between ageing and cancer. We also need to further investigate age‐related patterns in the tissues and underlying mechanisms of tumour growth and dissemination. For example, in colorectal cancer, the incidence and metastatic spread of which declines with age, further exploration is needed to decipher how ageing interacts with other parameters (such as with the exposome and genetic background) to modulate disease biology, in tumour cells and their microenvironment (e.g. through inflammaging, immunosenescence (Glossary, Box 2) and modified microbiota) [39]. More generally, exogenous factors related to lifestyle, dietary factors and toxic exposure together with internal factors (genetic background, associated diseases and microbiota) may modulate both ageing and the risk of cancer (Fig. 4). We also need to understand how ageing affects treatment efficacy and to what extent cancer and cancer treatment accelerate ageing, as discussed below.

Future research on ageing and cancer (Fig. 2A) may address

- The link between cell competition in ageing epithelia and early steps of cancer.
- The distinct paths to nonmalignant clones and to cancer.
- The boundaries between a nonmalignant and a malignant clone.
- The biological specificities of ageing‐associated cancers.
- The cooperation of ageing and other cancer risk factors.
- The ability of interventions aiming at slowing down ageing effects to prevent cancer.

## 3 Biological assessment of ageing effects

Frailty is common in older patients, making them more prone to the adverse outcomes of cancer treatment. In patients aged 65 and older, the ‘risks versus benefits’ balance can be weighed up by conducting a geriatric assessment (Glossary, Box 2) of patients for their functional, emotional and nutritional status, for their comorbidities and ongoing treatments, and for their cognition and social support [40]. Ideally, this geriatric assessment would ensure that the patient’s treatment is co‐ordinated and managed by an interdisciplinary health care team, including hospital‐ and home‐based professionals, who incorporates into a personalized care plan the patient's goals and preferences. Nononcological interventions such as pre‐habilitation care (Glossary, Box 2) [41] can be requested from this geriatric assessment to support cancer resilience. Recently developed web‐based apps can also be used to record medical data, and self‐management guidelines can be provided to patient to prevent avoidable adverse events. In reality, however, implementation of geriatric assessment and management into daily oncology practice remain difficult in many places for multiple reasons [40].

Chronological age (Glossary, Box 2) alone is a poor indicator of the physiological and functional status of older cancer patients. The quantification of ageing biomarkers to estimate an individual’s biological age (Glossary, Box 2) is an actively developing field. This research in biohorology measures the passage of time in living systems. It aims to provide better metrics that can correlate with various clinical parameters as compared to generalized health information associated with a particular age range [42]. The disparity between predicted and actual age, also referred to as ‘delta age’, might better reflect an individual’s overall health [43].

Molecular footprints of ageing include methylated DNA [44, 45, 46], gene expression profiles [47, 48], circulating proteins [49, 50], metabolites [51, 52], biochemical markers [53, 54] and microbiota [55]. In the last decade, these composite analyses have generated a multitude of ageing clocks that reflect different aspects of ageing and that identify organ‐specific temporal signatures [56, 57] and correlate with various health outcomes. One such clock is exemplified by the methylation of a subset of age‐related CpG sites on DNA. When the methylation rate of each of these sites was analysed computationally, researchers found this measure to provide an accurate biomarker of age in humans [40]. The observed synchronicity that exists in the organs despite different proliferation dynamics points to the hypothesis of a master pacemaker of epigenetic age acting on tissues via blood‐borne regulatory factors [58].

Systemic factors are powerful regulators of ageing [59]. For example, young mice exposed to the blood of old mice via heterochronic parabiosis (Glossary, Box 2) exhibit decreased synaptic plasticity as well as impairments in memory and learning [60, 61]. It has also been reported that levels of proteins that are implicated in the innate and adaptive immune systems in humans, and that regulate life span in animal models, accurately predict age [62]. Importantly, individuals who are predicted to be younger than their chronological age performed better on physical and cognitive tests [49]. Further refinement of these analyses can be anticipated, for example, through advanced quantitative proteomics analysis exploring the link between dynamic changes in post‐translational protein modifications and age‐associated diseases such as cancer [63].

Each of these approaches has technological limitations, and the ageing clock concept is still nascent. For example, different proteomics and ageing studies have used different proteomics techniques, patient populations, statistical analyses, tissues and cell types, and their findings significantly vary from one another [19, 64, 65]. It remains largely unknown if the inputs used to determine biological age represent drivers of function or downstream effects. Finally, much work has yet to be done to confidently determine whether these applications can be translated in clinical oncology. Machine learning modelling and neural networks (Glossary, Box 2), using SHAP (SHapley Additive exPlanations) and other approaches to explain the output of the models [66], might be the next power tools to develop this appealing approach. They could, for example, enable more precise and earlier diagnosis of cancer [67, 68]. Biological age and ageing clocks also have the potential to guide therapeutic decision, to indicate anti‐ageing intervention [69] and, through repeated measurement, to evaluate the effects of cancer treatment on biological age [45]. Thus, serial measurement of biological age taken from safely and easily obtainable samples (such as from blood, urine and saliva) might be highly informative on an individual level, before and after cancer treatment.

Future research on biological age measurements (Fig. 2B) may address

- The best approach to measure biological age in routine clinics.
- The link between biological age biomarkers and cancer risk.
- The performance of biological age measurements as compared to geriatric assessment.
- The ability of biological age measurements to guide anticancer treatment choices.
- Their contribution to the monitoring of individual’s response and survivorship.

## 4 Muscle mass and sarcopenia

Another common effect of ageing, which is associated with adverse outcomes in oncology, is the linear loss of muscle mass described as sarcopenia. Muscle mass decreases with age, beginning as early as the fourth decade, while body fat gradually increases until the seventh decade of life, sometimes leading to sarcopenic obesity [70]. In mice, exercise has been shown to improve the functionality of muscle stem cells, to restore nicotinamide adenine dinucleotide (NAD^+^) metabolism by slowing down age‐related decline in nicotinamide phosphoribosyltransferase (NAMPT) [71] and to increase the capacity of aged muscle to repair injury [72].

In the oncology field, total body skeletal muscle and adipose tissue have been commonly estimated by using a single abdominal computed tomography cross‐sectional image, which has been shown to be an independent determinant of chemotherapy toxicities [73] and of surgical outcomes [74]. To be accurately measured and explored, a consensus definition of sarcopenia is needed in order [70]. The oncology field focussed on the association of low muscle mass with an increased incidence of side effects of cancer treatments and mortality. Since low muscle mass is also observed in ~ 25% of adults younger than 60 years who are suffering from cancer, and given that cancer treatment can accelerate muscle mass loss, more research is needed to distinguish the mechanisms of sarcopenia in ageing and cancer patients.

Sarcopenia could also be targeted by nononcological interventions. For example, a bone to muscle feed‐forward endocrine axis involving the bone‐derived hormone osteocalcin has been shown to reverse the age‐induced decline in exercise capacity in mice. Circulating levels of osteocalcin dramatically decrease in middle age and double during aerobic exercise. Exercise capacity of aged mice increases when they are injected with this hormone, fully restoring muscle function and increasing muscle mass [75]. Osteocalcin is only a part of the story: multiple molecular mechanisms cause for sarcopenia, including hormones, muscle fibre composition, myo‐satellite cell potential to differentiate and proliferate, inflammatory pathways, intracellular proteostasis and mitochondrial functions [76].

Future research on sarcopenia (Fig. 2C) may address

- The best definition and measurement of sarcopenia in cancer patients.
- The metabolic and inflammatory changes that lead to sarcopenia.
- The mechanism of muscle loss differs in ageing patients with and without cancer.
- The efficient interventions to correct or prevent sarcopenia in older patients with cancer.

## 5 Cancer prevention in ageing people

Prevention‐based approaches might offer the most promise when it comes to ageing interventions [77]. One approach, called passive prevention, is based on risk avoidance long before ageing occurs such as not smoking tobacco. Another approach called ‘interception’, as first named by Elisabeth Blackburn [78], is based on the idea that, as ageing‐associated cancers typically develop over years and sometimes decades, it might be feasible to actively intercept a malignant tumour before full‐blown clinical expression.

Such an ‘interception’ strategy was proposed in the context of genetic predisposition, the idea being to treat an individual before the clinical detection of a tumour, using a drug that is effective in treating an established cancer. Examples of those include using an oral smoothened (SMO) inhibitor to prevent basal cell carcinoma in individuals with Gorlin syndrome [78, 79] or the use of a poly(ADP‐ribose) polymerase (PARP) inhibitor to prevent cancer in individuals with *BRCA*1 mutation [80]. This interception approach needs to be carefully evaluated, however, to detect the potentially deleterious effects of these drugs when used in a preventing setting.

In ageing‐related cancers, a similar active cancer interception approach would aim to decrease the number of senescent cells that accumulate in all ageing organs and disorganize tissue architecture [22]. Targeting senescent cells might not only prevent cancer but also mitigate other chronic diseases by reducing chronic inflammation generated by the SASP [81].

Novel pharmaceutical interventions that aim to interfere with the detrimental effect of senescent cells, either by eliminating (senolytics) or modifying (senomorphics) senescent cells (Glossary, Box 2), are entering the clinical stage [1]. Despite the lack of universal and specific markers of senescence, and the specification of several senescence types, strategies are being developed to better identify senescence‐associated phenotypes in order to closely monitor the efficacy of an anti‐senescence therapy [82]. Such ageing interventions, sometimes compared with hardware defragmentation, might benefit from the exponential growth of pharmacological approaches (see, for example, the DrugAge database of ageing‐related drugs at https://genomics.senescence.info/) and the appearance of an anti‐ageing biotech sector. *In silico*‐based screening approaches could also be developed to identify more rapidly potentially active molecules, given the time and costs of validating these interventions [83].

Additional cancer prevention strategies might also emerge from the better identification of specific ecosystems that impact cancer emergence, either in individuals (such as genetic and epigenetic alterations, disabilities and socioeconomic disadvantages) or in populations (such as exposure to chemicals, pathogens and radiations). As of today, it remains unclear if rejuvenating strategies that eradicate nonmalignant clones in healthy tissues would preserve their tumour suppressive properties and decrease the risk of cancer development or whether they would suppress a protective mechanism [18].

Multiple questions need to be answered to increase our ability to prevent ageing‐related cancer. The analysis of genetically engineered preclinical models might help us to decipher the main functional, genetic or epigenetic routes that drive ageing‐related cancer initiation. Collecting data from humans as they age and analysing it with machine learning tools is also an important approach with which to explore single‐patient disease trajectories across decades. They might also help us to predict, and, when possible, to prevent ageing‐associated diseases including cancer [67, 84, 85]. The development of innovative approaches such as minimally invasive liquid biopsies to detect the appearance of circulating premalignant cells and cell‐free DNA [86], together with technological advances in clinical imaging [87], will further enrich these approaches (see the following discussion on early diagnosis). Finally, the use of artificial intelligence‐powered preventive medicine strategies in longevity medicine could help us to understand whether an individual is ageing faster and, if so, how to slow down its effects [43].

Future research on cancer prevention in ageing people (Fig. 2D) may address

- The improvement of the monitoring of senescent cells in ageing tissues.
- The use of AI‐powered preventive strategies to slow down ageing effects on tissues.
- The safe interventions that could prevent ageing‐associated diseases including cancer.
- The prevention programmes that could avoid secondary cancer when ageing.

## 6 Early diagnosis of cancer in ageing people

Population‐based screening for early cancer detection within specific age intervals has been implemented in most European countries, usually between the age of 40 and 74 years for breast and colorectal cancers. These screening programmes ensure informed choice, confidentiality and respect for autonomy while promoting equity and access to screening for the entire target population. In older individuals, the benefits of population‐based cancer screening are more uncertain. The harms of such screens, which typically include over‐diagnosis and complications from downstream diagnostic interventions, increase with age. For example, the balance between breast cancer screening benefits and harms becomes less favourable after 74 years of age and, at age 90 years, the harms outweigh the benefits, largely as a consequence of over‐diagnosis [88]. The scarcity of adults older than 75 years in controlled clinical trials is a further barrier to the generation of scientific evidence of the effectiveness of screening programmes in this population. A current recommendation is to individualize cancer screening for older adults by accounting for life expectancy, comorbidities, individual values and the risks and benefits of specific cancer screening tests [89].

Population‐based and individual screening approaches have, so far, mostly relied on conventional diagnostic methods with physicians using visual pattern recognition to identify concerning lesions detected clinically or radiologically. The landscape will likely change with the development of artificial intelligence that has the potential to revolutionize the early diagnosis of cancer, based on the use of machine learning algorithms that can analyse large volumes of cancer diagnosis data [68, 90]. This approach, based on multi‐layer neural networks in which statistical methods are used to train data to automatically adjust the parameters of a model, improves diagnosis performance with increasing experience and data. Machine learning algorithms can be supervised, where they rely on the use of labelled data, or unsupervised, where they detect hidden patterns that cannot be detected by humans. In cancer diagnosis, machine learning is supervised, as the goal is to classify digitized clinical or radiological images into predefined categories, such as benign or malignant [91].

The computational analysis of the early steps of oncogenesis might improve the timely detection of early‐stage cancers as machine learning tools can incorporate molecular information [92]. As proposed by the LifeTime Initiative (https://lifetime‐initiative.eu) [93], the analysis of single‐cell multi‐omics and images is needed to characterize cell types (transformed cells and their environment) and cell states in early‐stage cancers. This data can be collected longitudinally from patients and from patient‐derived experimental models during the progression from health to disease [87]. Machine learning techniques can also be used to analyse data obtained from liquid biopsies. This minimally invasive test uses blood samples to look for cancer cells or pieces of DNA from tumour cells present in the blood. This search can be extended to the analysis of miRNAs, epigenetic changes and extracellular vesicles, as well as to the detection of premalignant lesions. It can also be used to analyse other biological liquids, such as urine and saliva [86, 94, 95]. Liquid biopsy is also a comprehensive approach to evaluating tumour heterogeneity since every tumour site can release aberrant signals into body fluid. Ongoing work will indicate the extent to which this approach can contribute to early cancer detection strategies for older individuals, especially in the context of population‐based screening [96].

Following the appropriate generation and analysis of data, the development of machine learning and of minimally invasive screens should improve the early detection of cancer. They should also drive an interceptive approach that aims to eradicate early‐stage cancers.

Future research on early diagnosis of cancer in ageing patients (Fig. 2E) may address

- The collection of images and molecular data of early‐stage cancers in ageing patients.
- The hidden patterns of early‐stage cancers in clinical images and molecular data.
- The development of algorithms to improve older population‐based screening of cancers.
- The generation of minimally invasive tests for screening cancer in older individuals.

## 7 Improving the treatment of established cancers in ageing patients

There is a persistent mismatch between the age of cancer patients included in clinical trials and that of individuals who are most likely to get cancer, indicating a need to redesign clinical trials to address this concern [97]. A survey of patients who had been enrolled onto US National Cancer Institute adult trials between 2001 and 2011 showed that < 25% of them were aged 65 or older, and < 10% were aged 75 or older with no significant increase in the recruitment of these age groups over the time [98]. Barriers to the participation of older patients in clinical trials are particularly marked at 80 years or older [99]. The inclusion of more older patients in randomized controlled trials better explores the risk–benefit ratio for cancer treatments as a function of age [100, 101, 102].

The geriatric assessment of aged individuals by a multidisciplinary team to address pro‐actively all of their individual frailties should be more commonly implemented before any treatment is initiated in geriatric oncology practice. And, more randomized controlled trials must explore the benefit of interventions, as guided by standardized geriatric assessment [40], in terms of toxicity reduction without compromising survival benefits.

In one such trial involving more than 600 patients with diverse solid tumours, geriatric assessment was provided to treating oncologists and compared with the interventions implemented by a geriatrics‐trained multidisciplinary team. The trial demonstrated a significant reduction in grade 3 or higher chemotherapy‐related toxic effects in older adults with cancer who had undergone a geriatric assessment [103]. One study showed that in patients aged 70 years and older, whose oncologists received a tailored geriatric assessment summary and management recommendations, toxic effects of their cancer treatment were significantly reduced as compared to usual care [104]. Ongoing randomized trials will provide further evidence‐based interventions following geriatric assessment [105].

A large retrospective cohort study has also assessed the postoperative outcome of cancer patients aged 75 years and older. This study observed a significantly lower 90‐day postoperative mortality among those who received geriatric assessment‐driven care versus those who had not [106]. However, a prospective randomized trial testing the impact of perioperative geriatric intervention in patients undergoing surgery for a gastrointestinal cancer did not confirm a significant impact of this intervention on postoperative hospital length of stay, intensive care unit use, hospital readmission and complications, indicating the need to further refine this strategy [107]. There remains a need to pursue the implementation of geriatric assessment in routine surgery practice and to improve the subsequent geriatric care. This approach should aim to define how pre‐operative geriatric assessment can guide the use of minimally invasive approaches and, when combined with tailored recovery protocols, how this assessment can reduce surgical stress and promote functional recovery [108].

Over the two last decades, targeted therapies and immunotherapies that use immune checkpoint inhibitors (ICIs) are being increasingly used in cancer care. As many of these new drugs have a favourable toxicity profile as compared to cytotoxic chemotherapy, they might be offered to frail older cancer patients who may not have previously been offered this type of cancer‐directed therapy. However, ageing‐associated changes such as immune‐senescence require specific attention in this patient population [109]. Available evidence indicates that the clinical efficacy and toxicity of most of these drugs is comparable in fit older and younger adults included in clinical trials. By contrast, their effectiveness and toxicity among frail older adults is still poorly understood as these patients were excluded from the landmark clinical trials [110, 111, 112]. More inclusive clinical trials have now been designed to specifically address how immunosenescence in frail patients could alter the therapeutic response to ICIs or increase their side effects (as with NCT04533451). In addition to the harms identified in younger patients, these clinical trials will evaluate the impact of new drugs on the functional status and quality of life of older patients.

There is much to learn from ageing biology that could also benefit to younger patients. One of the mechanisms by which cancer therapies exert antitumour activity is by inducing tumour cell senescence. In clinical medicine, in addition to inducing cancer cell death, radiation and chemotherapy cause the accumulation of senescent cells in the tumour and, in most cases, in the surrounding healthy tissues. The accumulation of these senescent cells in a tumour and its environment can, paradoxically, promote tumour relapse, metastasis and resistance to therapy, in part, through the expression of the SASP. Senescent cells in normal tissues that surround the tumour also contribute to radiation‐ and chemotherapy‐induced side effects. In the ‘one‐two punch’ approach to treating cancer, therapeutics aim to induce tumour cell senescence, followed by the selective clearance of senescent cells. According to this concept, senolytic drugs such as inhibitors of the pro‐survival proteins of the Bcl‐2 family could be used to convert senescent cells into dead cells and thus maximize cancer treatment efficacy, preventing relapse while reducing the acceleration of ageing induced by these treatments in cancer survivors [113, 114].

Future research on the treatment of established tumours in older patients (Fig. 2F) may address

- The generation of evidence‐based guidance.
- The risk–benefit ratio for cancer treatment as a function of age.
- The benefits of geriatric assessment‐ and biological age‐driven individualized interventions.
- The benefit of robotic and minimally invasive surgery over classical surgical procedures.
- The prevention of cognitive dysfunction induced by cancer treatment.
- The combination of senolytic drugs with chemotherapy and radiation therapy.

## 8 Survivorship in ageing patients treated for cancer

As a consequence of population growth and ageing as well as of advances in cancer early detection and treatment, the number of cancer survivors is increasing. A recent survey in the United States indicates that 56% of survivors were diagnosed within the past 10 years and 64% are aged 65 years or older [115]. Older cancer survivors have unique medical and psychosocial needs that concern cancer‐ and treatment‐related short‐term and long‐term health effects, yet they remain under‐represented in survivorship research (Glossary, Box 2).

The first specific need of this population is for clinical oncologists to incorporate pretreatment geriatric assessment (integrating age, comorbidities and cognitive and physical impairments) in routine practice. This will help to better predict the feasibility, safety and efficacy of interventions in this patient group. For those patients most likely to experience therapy‐induced toxicity, research is needed to generate evidence‐based guidelines for how to adapt therapeutic schemes and objectives. Guidelines are also needed for nononcological interventions that could preserve physical performance, nutritional status and cognition in cancer survivors. Such evidence‐based decisions will have to be stratified by cancer type. The weighting of composite endpoints including reduced toxicity and maintained functions may be as important as improving survival in older patients. Research should also move rapidly beyond geriatric assessment and explore how measurements of biologic age, for example, by using minimally invasive techniques [54], evaluation of sarcopenia [70], detection of cellular senescence [82, 109] and biomarkers of inflammation [116], could measure frailty, improve the prediction of outcomes and better guide therapeutic intervention.

When adjusted for age, sex and lifestyle factors, cancer survivors are more likely to suffer from chronic diseases, including second cancers, than are their age‐matched counterparts, possibly due to accelerated biological ageing and treatment‐induced adverse events. Thus, not only is ageing a significant risk factor for cancer but cancer and its treatment also significant contribute to ageing, indicating a bilateral relationship [117, 118, 119]. Hallmarks of accelerated ageing include anatomical, functional and mechanistic effects that are similar to those of ageing but are detected at a younger age than usual [120]. Whether cancer by itself promotes ageing or is a marker of ageing remains a controversial issue. In contrast, many modalities used to treat cancers damage healthy tissue, either accentuating or accelerating the ageing process. Chemotherapy and radiation therapy carry a ‘gerontogenicity’ potential through free radical formation, DNA damage and telomere shortening [121]. Although paediatric cancers have different underlying mechanisms, exploring long‐term outcomes in childhood cancer survivors may provide some insights into this issue.

Another well‐known effect of chemotherapy and radiation therapy for a primary cancer is the induction of a second cancer, especially a therapy‐related myeloid neoplasm [122]. These neoplasms have long been thought to develop from the mutagenic effects of cancer therapy, with alkylating agents, topoisomerase inhibitors and poly(ADP‐ribose) polymerase inhibitors [123] inducing genomic alterations in haematopoietic stem and progenitor cells through diverse mechanisms. Murine models and clinical observations both suggest that, in addition to mutagenic effects, cancer treatment can shape the selection of clonal mosaicism (Glossary, Box 2), meaning that they can increase the fitness of pre‐existing clones [124, 125]. In such a situation, clonal mutations detected in peripheral blood cells before cancer treatment are the initiating events of a therapy‐related myeloid neoplasm. Clonal mutations in the DNA damage response genes *TP53*, *PPM*1D and *CHEK2* are most specifically associated with these therapy‐related myeloid neoplasms. Among treatment modalities, radiation and cytotoxic therapies (mostly topoisomerase II inhibitors and platinum agents) are again strongly associated with clonal hematopoiesis evolution, an event that is not observed when targeted therapies and immunotherapeutic agents are used [126, 127]. In the near future, screening for clonal hematopoiesis may form part of risk stratification before cancer treatment, leading to the adaptation of a therapeutic strategy. This strategy would ideally include the interception of high‐risk clones to prevent subsequent myeloid neoplasm.

In addition to these biology‐driven approaches [128], improving the survivorship of older cancer patients may require a personalized survivorship care plan that includes lifestyle guidance tailored to the patient’s needs such as on exercise, nutrition, polypharmacy, social support and comorbidities, together with careful follow‐up following a surveillance plan. A research objective will be to validate the format and outcomes of such plans, testing different models of care with interdisciplinary teams coordinating survivorship care delivery.

Future research on the survivorship of older cancer patients (Fig. 2G) may address

- The mechanisms by which cancer and cancer treatment promote ageing.
- The role of pre‐existing clonal hematopoiesis in therapy‐related leukaemia.
- The best predictor of quality of life and biological resilience in older cancer survivors.
- The optimal, evidence‐based survivorship care plans.
- The prevention of social exclusion of cancer survivors.

## 9 Health disparities among older cancer patients

Attempts at measuring health disparities and at ensuring health equity for older adults with cancer can be challenging. In addition to differences between age groups, the origins of health disparities such as differences in socioeconomic status, sex, geography or ethnic groups have to be explored within each age group [129, 130]. Some health disparities have biological origins, for example, mitochondrial determinants contribute to disparities in cancer susceptibility and severity among ethnically different populations [131, 132]. Nevertheless, better understanding of these biological disparities could help to generate innovative approaches by reducing their impact on cancer prevention and treatment. Beyond biology, the highest cause of inequities in the health of older patients is related to the sociodemographic index [133]. Socioeconomic disparities generate lifelong (dietary, lifestyle and exposome; Glossary, Box 2) disparities that may accelerate tissue ageing. In turn, tissue ageing will cooperate with other risk factors to promote cancer development.

**Fig. 5:**
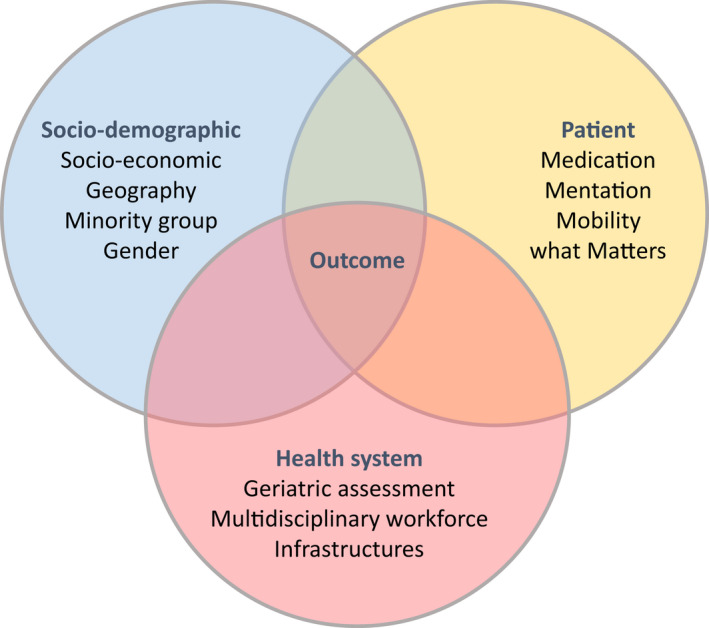
Disparities influencing outcome of older patients with cancer.

To ensure health equity among older cancer patients while facing the ageing of the population and the increased survival of cancer patients, we need to increase the number of oncologists with geriatric expertise. Such an objective requires training programmes in combined geriatrics and medical oncology. An increased workforce in geriatric oncology could drive the implementation of age‐friendly health systems in which individualized assessment of the 4Ms (what Matters to the patients and their family, Medications, Mentation and Mobility) could be provided to each patient to guide individualized plans of care [134]. Finally, health equity requires that we ensure that minority groups are adequately considered among older adults, for example, equally included in clinical trials [2, 135] (Fig. 5).

The time and resources needed to train and recruit this additional workforce may further increase inequalities among world regions, for example, among European member states, which have diverse income and sociodemographic indices. The complexity of cancer care in ageing patients magnifies the usual pitfalls in the optimal delivery of health care, including financial, logistical and medical hurdles. Optimizing the treatment of cancer and the quality of life of survivors in older patients requires both public and policymaker support. To build this support, we need to disseminate evidence‐based policies in understandable and compelling ways. We also need to solve conflicts between the common principles of equity and the use of noncost‐effective cancer treatments [136].

Future research on health disparities among older cancer patients (Fig. 2H) may address

- The biological bases of inequities among older individuals facing cancer.
- The optimal training of geriatric caregivers and oncologists.
- The use of social network tools to improve communication between patients and caregivers.
- The maintenance of social links between ageing cancer patients, society and health authorities, including their contribution to programme funding decision.
- The broadly acceptable definition of value in a cancer treatment applied to older patients.

## 10 Conclusions

The spectacular progress in our understanding of the biology of ageing in recent decades, together with the increase in ageing population worldwide, the rising incidence of cancer, and the rising efficacy and cost of cancer treatments, justify expanding our research on cancer and ageing. This research, based on the suspicion that ageing is a modifiable cancer risk factor, might deliver evidence‐based recommendations to improve the prevention, early diagnosis, care and survivorship of older patients with cancer (Fig. 2). Five main biological targets have been identified to reduce the effects of ageing. They include the insulin growth factor pathway, the mammalian target of rapamycin (TOR) pathway, the family of sirtuins, the mitochondria and cells undergoing senescence. Most of these targets can also interfere with cancer biology [1], suggesting that modifying ageing could also target emerging malignant cells. For example, metformin could favourably interfere with both ageing effects and cancer [137].

The increasing burden of cancer in older patients requires that we train a new generation of geriatric oncologists and researchers to address the complexity of the disease in a multidisciplinary approach. The development of geriatric assessment‐guided interventions is increasingly used to generate evidence‐based recommendations. When cancer is there, age by itself does not fully characterize the physiological heterogeneity of patients and so, we also need to develop novel biomarkers that can provide precise information on an individual’s biological age. With this information in hand, the outcome of choice for cancer treatment in older patients must be carefully evaluated with each individual patient to decide whether survival by itself is more meaningful than functional independence and quality of life.

## Conflict of interest

The authors declare no conflict of interest.

## Author contributions

ES wrote a draft of the manuscript that has been reviewed and corrected by NAZ and FC.
